# Editorial: Global excellence in ethnopharmacology: Asia

**DOI:** 10.3389/fphar.2023.1298718

**Published:** 2023-10-23

**Authors:** Somasundaram Arumugam, Aiping Lyu, Bey Hing Goh, Uraiwan Panich

**Affiliations:** ^1^ Department of Pharmacology and Toxicology, National Institute of Pharmaceutical Education and Research (NIPER)-Kolkata, Kolkata, West Bengal, India; ^2^ School of Chinese Medicine, Hong Kong Baptist University, Kowloon, Hong Kong SAR China; ^3^ Sunway Biofunctional Molecules Discovery Centre (SBMDC), School of Medical and Life Sciences, Sunway University, Sunway, Malaysia; ^4^ College of Pharmaceutical Sciences, Zhejiang University, Hangzhou, Zhejiang, China; ^5^ Biofunctional Molecule Exploratory Research Group (BMEX), School of Pharmacy, Monash University Malaysia, Bandar Sunway, Malaysia; ^6^ Department of Pharmacology, Faculty of Medicine Siriraj Hospital, Mahidol University, Bangkok, Thailand

**Keywords:** ethnopharmacology, Asia, innovation, trad. chinese medicine (TCM), ayurveda

## Introduction

Ethnopharmacology is a multidisciplinary field combining elements of anthropology, pharmacology, botany, chemistry, and other disciplines to study various cultures’ traditional knowledge and medicinal use of plants, animals, and other natural substances (Reyes-García, 2010). It focuses on understanding the relationship between different cultural groups and the plants and substances they use for healing and investigating the scientific basis for the efficacy of these traditional remedies (Fokunang et al., 2011). Ethnopharmacologists study the traditional healing practices of different cultures, including indigenous and local communities. They document the use of specific plants, animals, minerals, and other natural substances in treating various ailments, including botanical and chemical analyses to determine the active metabolites responsible for therapeutic effects. Ethnopharmacologists also focus on conserving medicinal plants and other natural resources used in traditional medicine by promoting sustainable harvesting and cultivation practices to ensure the availability of these resources for future generations. Traditional remedies discovered through ethnopharmacological research can serve as sources of inspiration for developing new pharmaceutical drugs. Most modern medications can be traced back to their origins in traditional remedies. Hence, the established credentials and credibility of ethnopharmacology are widely acknowledged and can contribute to integrating traditional medicine into modern healthcare systems, particularly in areas where traditional remedies are extensively utilized. This integration has the potential to enhance healthcare accessibility and mitigate healthcare disparities. Keeping this in mind, this topic has been chosen to have a collection of ethnopharmacology-based research in Asia, which has the richest history of several traditional medicine systems.

## Ethnopharmacology in Asia

*Astragalus membranaceus* Bunge (Family: Fabaceae), commonly known as Huangqi or Astragalus, is a prominent botanical drug in Traditional Chinese Medicine (TCM) with a long history of use. In TCM, Astragalus is considered a powerful Qi (body’s vital energy or life force) tonic. It is frequently used to combat fatigue, boost energy levels, and improve physical endurance. It supports the immune system in addition to its adaptogenic properties. It has anti-inflammatory effects and promotes cardiovascular health by improving blood circulation, reducing blood pressure, and protecting the heart from various forms of stress. Some research suggests that Astragalus may help regulate blood sugar levels and improve insulin sensitivity. Astragalus is often associated with longevity and anti-ageing properties in TCM. It improves digestive health and promotes overall health and wellbeing (Wang et al.).

Similarly, Wen et al. have reviewed the chemical composition, pharmacology, pharmacokinetics and toxicity of *Acorus calamus* var. *angustatus* Besser (Family: Acoraceae) rhizome, a natural product commonly known as “Shi Chang Pu” or “Acori tatarinowii rhizome” in TCM. In TCM, Acori tatarinowii rhizome is classified as a botanical drug that “opens the orifices” and is known for its ability to clear the mind, improve mental clarity, and enhance cognitive function. It also has sedative effects, making it useful for anxiety, insomnia, and restlessness. This botanical drug is also used in TCM to address digestive issues, including abdominal pain, diarrhoea, and bloating. Precaution is necessary since *Acori tatarinowii* contains certain metabolites, such as β-asarone, which can have toxic effects when consumed in large quantities.

Geng et al. reviewed the ethnobotany, phytochemistry and pharmacological properties of *Fagopyrum cymosum* (Trevir.) Meisn. (Family: Polygonaceae), also known as “He Ye” or “*Fagopyri dibotryis* Rhizome” in TCM. It is traditionally used to clear heat and toxins from the body in conditions associated with excessive heat, such as fever, sore throat, and skin rashes. Because of its anti-inflammatory properties, it is helpful in inflammatory skin disorders. Some traditional uses of *F. dibotryis* rhizome involve its diuretic properties. Experiments performed *in vitro* and *in vivo* showed that the extracts or fractions of *F. dibotryis* rhizome had a wide range of pharmacological activities, including antitumor, anti-inflammatory, immunomodulatory, antioxidant, antimicrobial, and antidiabetic. They have concluded that *F. dibotryis* rhizome is worthy of further study and application as a potential antitumor drug.

Apart from these reviews, this topic has five research papers focusing on various traditional or ethnomedicine and their pharmacological activities. Choudhary et al. have studied the antidepressant effects of a natural coumarin antioxidant, 4-methyl esculetin, that is isolated from the peels of *Aesculus hippocastanum* L (Family: Sapindaceae), through the inhibition of LPS-induced NLRP3 inflammasome activation. They have performed *in silico*, *in vitro* and *in vivo* studies and showed the antioxidant, anti-inflammatory and antidepressant effects, possibly through the inhibition of NLRP3 inflammasome activation, suggesting the potential clinical use of 4-methyl esculetin in NLRP3 inflammasome-associated inflammatory diseases such as depression.

The comprehensive metabolomic profiling analysis combined with network analysis of serum pharmacochemistry has revealed the therapeutic mechanism of *Ardisia japonica* (Thunb.) Blume (Family: Primulaceae) also known as “Ardisiae Japonicae Herba (AJH)” against acute lung injury in LPS-induced rats (Han et al.). Metabolomics results showed that AJH effectively treated ALI by alleviating the infiltration of inflammatory cells in alveolar spaces and regulating the expression of inflammatory cytokines. AJH might link to reverse the abnormality of phenylalanine, tyrosine, and tryptophan biosynthesis and linoleic acid metabolism pathways to regulate the concentrations of potential biomarkers to normal levels. Therefore, AJH could alleviate inflammation responses in the Acutlung injury treatment. A similar study evaluated the molecular mechanism of Yinchen Sini decoction in CCl_4_-induced acute liver injury in mice using Integrated network analysis and metabolomics (Zheng et al.).

A medical ethnobotanical assessment was conducted to comprehensively understand the single botanical drugs in Ayurvedic Pharmacopoeia of India (API) (Yao et al.). They also highlighted the importance of quantitative ethnobotanic methods in understanding traditional medical knowledge. They have clarified the single botanical drugs, their biological origins, and their standard therapeutic uses. Their work provides ready-to-use medical ethnobotanical information on Ayurvedic botanical drugs.

The extensive exploration study of the traditional knowledge of the Gelao ethnic minority in North Guizhou, China, to gain an in-depth understanding of their botanical drug practices (Liu et al.). The field research in Gelao communities of Daozhen, Wuchuan and Zheng’an counties was done using interviews, surveys and participatory rural appraisal. Though this study had some limitations, including a lack of chemical and pharmacological analysis of the plants to verify their active metabolites and mechanisms of action, they have collected significant information on 187 medicinal plants, including their botanical names, sources, processing methods, primary therapeutic uses, and administration techniques. They also planned to conduct comprehensive and systematic investigations into Gelao traditional medicine and culture, establish appropriate protective measures, and develop high-value pharmaceutical products.

The application of digital intelligence technology in Processing Chinese Materia Medica (PCMM) was analyzed and discussed by Zhang et al. They have addressed the application of digital intellectualization technology in the processing of TCM, which promoted the standardization of the processing, the standardization of the quality of decoction pieces, the digitalization and intellectualization of production, and the safety and effectiveness of clinical use of TCM decoction pieces in addition to providing a theoretical basis for the technical progress and high-quality development of TCM industry in the future.

While valuable in many ways, traditional medicine systems have drawbacks and limitations, such as lack of scientific evidence, limited standardization, safety concerns, inadequate regulation, cultural and geographic limitations, resistance to change, limited documentation, limited accessibility, and lack of integration. It’s essential to recognize that traditional medicine can offer valuable insights into treating specific conditions and complement modern medicine. However, addressing these drawbacks through proper research, regulation, and integration is crucial to ensure the safety and efficacy of traditional medicine practices. One such approach is the recent establishment of guidelines for Phytopharmaceuticals to improve the quality of botanical drugs, though it applies only to plant-based products.

## Conclusion

In summary, ethnopharmacology serves as a vital link between traditional wisdom and contemporary scientific exploration. It plays a pivotal role in substantiating and harnessing the healing traditions from diverse cultures, simultaneously enriching our comprehension of natural remedies and their possible advantages. We trust that this Research Topic has comprehensively addressed significant research within the realm of ethnopharmacology and traditional medicinal systems, especially in the Asian context, which may inspire novel concepts and propel future research forward.
